# Identification of key genes associated with multiple sclerosis based on gene expression data from peripheral blood mononuclear cells

**DOI:** 10.7717/peerj.8357

**Published:** 2020-02-03

**Authors:** Zhenwei Shang, Wenjing Sun, Mingming Zhang, Lidan Xu, Xueyuan Jia, Ruijie Zhang, Songbin Fu

**Affiliations:** 1 Harbin Medical University, Laboratory of Medical Genetics, Harbin, China; 2 Harbin Medical University, Key Laboratory of Preservation of Human Genetic Resources and Disease Control in China, Ministry of Education, Harbin, China; 3 Harbin Medical University, College of Bioinformatics Science and Technology, Harbin, China

**Keywords:** Multiple sclerosis, Protein protein interaction networks, miRNA, SNP, Enrichment anclysis, Interaction

## Abstract

The aim of this study was to identify the potential key candidate genes of multiple sclerosis (MS) and uncover mechanisms in MS. We combined data from the microarray expression profile of three MS stages and performed bioinformatics analysis. Differentially expressed genes (DEGs) were identified among the distinct stages of MS and healthy controls, and a total of 349 shared DEGs were identified. Gene ontology (GO) and pathway enrichment analyses showed that the DEGs were significantly enriched in the biological processes (BPs) of purine-related metabolic processes and signaling, especially the common DEGs, which were enriched in some immunological processes. Most of the DEGs were enriched in signaling pathways associated with the immune system, some immune diseases and infectious disease pathways. Through a protein–protein interaction (PPI) network analysis and a gene expression regulatory network constructed with MS-related miRNAs, we confirmed FOS, TP53, VEGFA, JUN, HIF1A, RB1, PTGS2, CXCL8, OAS2, NFKBIA and OAS1 as candidate genes of MS. Furthermore , we explored the potential SNPs associated with MS by database mining. In conclusion, this study provides the identified genes, SNPs, biological processes, and cellular pathways associated with MS. The uncovered candidate genes may be potential biomarkers involved in the diagnosis and therapy of MS.

## Introduction

Multiple sclerosis (MS) is a neurodegenerative disease that affects the central nervous system (CNS) with autoimmune and inflammatory characteristics. The name multiple sclerosis refers to the numerous scars (also known as plaques or lesions) that develop on the white matter of the brain and spinal cord (Clanet, 2008). This disorder is characterized by the destruction of the myelin sheath, the material that surrounds and protects nerve cells. This damage slows down or blocks messages between the brain and body and leads to the symptoms of MS. MS is twice as common in women than in men and usually begins between the ages of 20 and 50 (Milo & Kahana, 2010). In 2015, approximately 2.3 million people globally were affected with rates varying widely in different regions and among different populations (GBD 2015 Disease and Injury Incidence and Prevalence Collaborators, 2015); in that year, approximately 18,900 people died from MS, which was increased from 12,000 in 1990 (GBD 2015 Mortality and Causes of Death Collaborators, 2015; GBD 2013 Mortality and Causes of Death Collaborators, 2013).

There are three clinical courses of MS. The most frequent is the relapsing-remitting form (RR-MS), which accounts for approximately 85% of MS cases. RR-MS is characterized by relapse followed by remission, where symptoms may vary from mild to severe, and relapses and remissions may last for days or months. After a variable time, most individuals with RR-MS advance to a secondary progressive phase (SP-MS), where neurologic worsening occurs without periods of remission. In contrast, 15% of individuals with MS experience a progressive course, called primary progressive MS (PP-MS), which is characterized by a steady worsening of neurologic functioning, without any distinct relapses or periods of remission. For PP-MS, the rate of progression may vary over time, with occasional plateaus or temporary improvements, but the progression is continuous (Confavreux & Vukusic, 2006).

The exact pathological mechanism of MS is unknown, but it is mostly regarded as an autoimmune attack of the myelin sheath, mediated by both cellular and humoral immunity. In recent years, several MS risk variants and susceptibility genes, such as rs6881706 in IL7R and rs6099045 in IL2RA, have been uncovered by genome-wide association studies (GWAS) (Beecham et al., 2013; Sawcer, Franklin & Ban, 2014). However, MS is a multifactorial disease determined by the complex interaction of genetic and environmental factors, whose integration occurs at the epigenetic level and determines gene expression. For example, studies show that smoking interacts with genetic risk factors such as HLA-DRB1*1501, HLA-A2, and NAT1 (Briggs et al., 2014; Hedstrom et al., 2011). In addition, Epstein-Barr virus (EBV) infectious mononucleosis, stress, and lack of vitamin D/sun exposure have been consistently assumed to be associated with an increased risk of developing MS (Olsson, Barcellos & Alfredsson, 2017).

Although, MS affects the CNS, there are evidences of altered immunity in the periphery in MS patients (Weiner, 2009). Further, the most widely used therapeutic drugs in MS are either immunosuppressive or immunomodulatory agents (Weiner, 2009; Wiendl & Hohlfeld, 2009), indicating that targeting peripheral immune system is beneficial to patients with this disease. These observations sustain the rationale for employing peripheral blood mononuclear cells (PBMCs) as an easily accessible and informative source of biological material in transcriptome studies for MS. Moreover many researches have recently shown the importance of blood transcriptomics in uncovering gene expression changes and transcriptional regulators in MS (Wiendl & Hohlfeld, 2009; Kemppinen et al., 2011; Zhang et al., 2011; Gilli et al., 2010; Christophi et al., 2008; Flores et al., 2003). In the present study, gene expression profile data from peripheral blood mononuclear cell (PBMC) samples of patients at different MS stages and controls were used to identify novel biomarkers and explore the occurrence and development mechanisms of MS. A variety of bioinformatics methods were applied systematically, such as differential expression analysis, GO and KEGG pathway enrichment analyses, PPI network analysis and sub-network analysis, and online database resources were also utilized. Finally, some significant results were obtained.

## Materials & Methods

The ArrayExpress database (https://www.ebi.ac.uk/arrayexpress/) of EMBL-EBI provides open access to high-throughput gene expression datasets (Parkinson et al., 2007; Brazma et al., 2003). The gene expression profile data from the E-MTAB-5151 dataset downloaded from the ArrayExpress database were analyzed in the present study. This dataset was established on the platform of A-AFFY-44-Affymetrix Gene Chip Human Genome U133 Plus 2.0 [HG-U133_Plus_2]. It contains 76 peripheral blood mononuclear cell samples, including 15 PP-MS, 21 RR-MS, 13 SP-MS and 27 healthy control samples. The patients with MS were diagnosed according to McDonald criteria6 and were not suffering from any other acute or chronic inflammatory diseases or other autoimmune disorders. Furthermore, they had not started any immunomodulatory therapy for MS yet. In the previous study, this dataset was used to validate the dysregulation of MS susceptibility genes (Srinivasan et al., 2017). The gene expression data of the three MS stages were thoroughly analyzed in our study to identify potential key candidate risk factors for MS.

### Data preprocessing and identification of DEGs

Background correction, normalization and probe summarization were performed on the original datasets, all the necessary preprocessing steps were performed using Robust Multiarray Average algorithm present in the Affy package in Bioconductor (http://www.bioconductor.org/packages/release/bioc/html/limma.html). Afterwards, the probes were mapped to genes, of which multiple probes with the same genes were merged to mean values. To identify the DEGs, linear models for the microarray data package Limma in Bioconductor was used. Moreover, considering the multiple-testing issues, in order to reduce the false positive rate of test results we have adjusted the results by FDR method, so the threshold of DEGs is FDR adjusted *p* < 0.01 and |log_2_fold-change (FC)| > 1. Differential expression analysis was conducted between the sample set of each MS stage and the sample set of healthy individuals, and the DEGs among the sample sets of the different MS stages were also identified.

### Gene Ontology (GO) and pathway enrichment analysis of DEGs

For the functional annotation of DEGs, the R package ClusterProfiler in Bioconductor was used (http://bioconductor.org/packages/release/bioc/html/clusterProfiler.html). This R package provides a comprehensive set of gene functional annotation tools to identify the enriched GO terms and KEGG pathways. Here we used “enrichGO” and “enrichKEGG” function that based on hypergeometric test to realize the enrichment analysis for DEGs sets. In this study, GO categories or KEGG pathways with FDR adjusted *p* < 0.05 were considered to be significant. In addition, the “dotplot” function in the package was used to plot the bubble diagram for showing the results of enrichment analysis.

### Analysis of Protein–Protein Interaction (PPI) network and module analysis

Research shows that some proteins work as monomers, but most of them work with chaperones or with other proteins. In order to analyze the possible role of Protein-Protein Interactions (PPIs) mapped by DEGs in MS pathogenesis, we constructed the MS related PPI network. In the present study, the common DEGs of three MS stages were mapped into Protein-Protein Interactions (PPIs) by using the Search Tool for the Retrieval of Interacting Genes/Proteins (STRING; https://string-db.org) database, which can provide information regarding predicted and experimental interactions of proteins. In addition to the interactions of experimental validation, we comprehensively considered other six types of evidence in predicting the associations of proteins, including the presence of fusion, neighborhood, cooccurrence, text mining, database evidence and co-expression. The interactions with a combined score >0.4 were selected as significant and retained. Then the MS related PPI network was constructed by Cytoscape software (version 3.4.0).

Hub genes are the genes that play a crucial role in the biological process, and in the related pathway, the regulation of other genes is often affected by these genes. Therefore, hub gene is often an important target and research hotspot. And in a network we can measure nodes by their network features scores to infer their importance in the network, and identify central elements of the network. In our study, the significant nodes and hub genes in the PPI network were obtained by using CytoHubba (http://www.cytoscape.org), a Java plug-in for Cytoscape (Chin et al., 2014). CytoHubba can rank nodes in a network by their network features, so as to identify significant nodes of the network. In our study, the nodes with higher Degree were considered as significant nodes. The plug-in Molecular Complex Detection (MCODE) was employed to screen network modules (Bader & Hogue, 2003), with established scores >5 and nodes >10 set in the MCODE module. Pathways with FDR-corrected *p* values less than 0.05 were considered statistically significant in the enrichment analysis of network modules. In addition, to explore the potential regulatory relationships and identify key risk factors of MS, we established a TF-miRNA-mRNA regulatory network of hub genes and MS-related miRNAs. For collecting MS-related miRNAs, we performed text mining in PubMed and retrieved databases such as the Mammalian NcRNA-Disease Repository (MNDR v2.0: http://www.rna-society.org/mndr/index.html) with a score>0.7 (mean strong correlation with MS), the Human MicroRNA Disease Database (HMDD v2.0: http://www.cuilab.cn/hmdd), and the miR2Disease database (http://www.miR2Disease.org). To obtain the related regulatory relationships of TF with miRNA, TF with gene and miRNA with mRNA, we retrieved information from the ChIPBase (http://rna.sysu.edu.cn/chipbase/) and starBase (http://starbase.sysu.edu.cn/) databases, and the regulatory relationships supported by 5 or more experiments were retained to construct the network.

### SNP analysis of the DEGs

To gain insight into the MS-associated SNPs, the common DEGs of different MS stages were subjected to SNP analysis. SNPs corresponding to these common DEGs were obtained from the online database SCAN (SNP and Copy number Annotation database; http://www.scandb.org/)  (Gamazon et al., 2010). We chose SNPs with frequencies greater than 0.10 and predicted the expression of these genes with *p* values less than 0.0001. A large number of SNPs corresponding to these common DEGs were obtained. However, to identify only the biologically significant SNPs, we sought these SNPs in the MirSNP database (http://bioinfo.bjmu.edu.cn/mirsnp/search/) (Liu et al., 2012), which can help identify SNPs present in the 3′ UTR of miRNA target sites. The mutation of these SNPs may affect the binding of the miRNAs to target genes, thereby affecting the expression of the corresponding genes. Then, with the list of MS-related miRNAs, we identified the most relevant SNPs that can be used for further screening and study.

## Results

### Selection of differentially expressed genes

**Figure 1:**
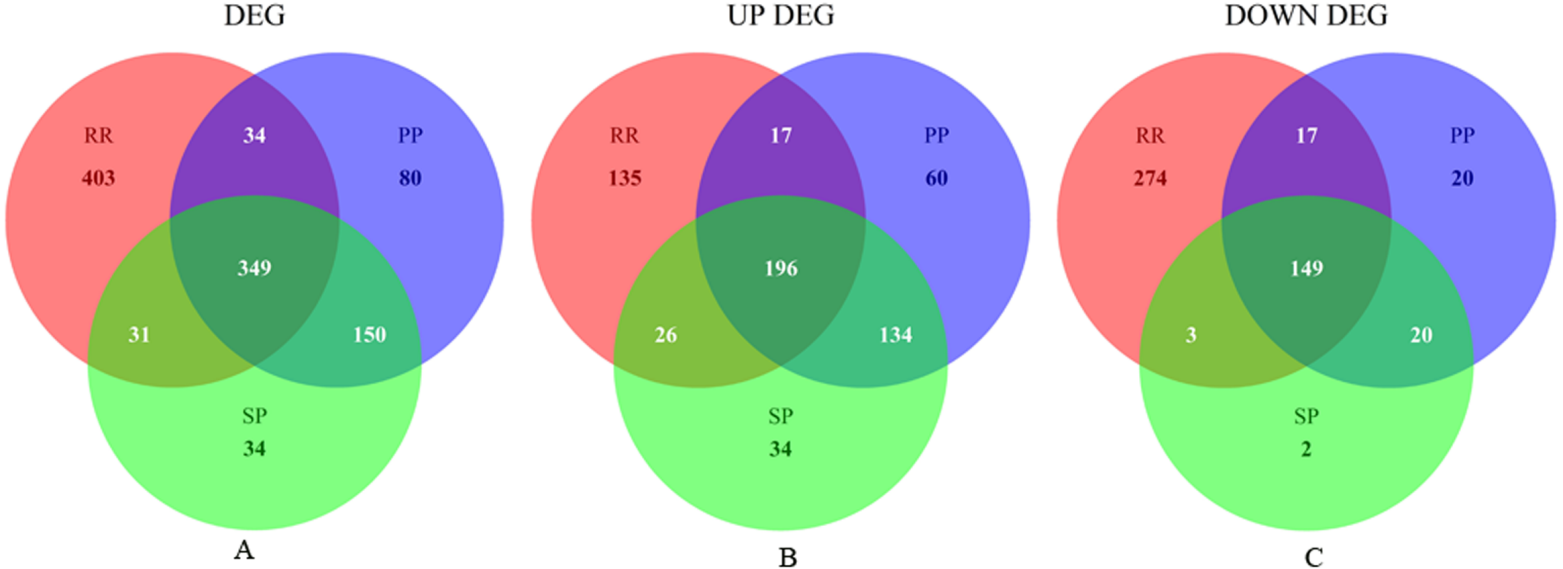
Venn plot of DEGs of three MS stages. (A), (B) and (C) respectively shows the Venn plot of dysregulated, up-regulated and down-regulated genes in three MS stages.

The present study analyzed the E-MTAB-5151 microarray data, containing 49 samples of three MS stages and 27 healthy controls. Following data normalization, preprocessing, differential gene expression analysis and filtering with the criteria of FDR adjusted *p* < 0.01 and |log_2_FC| > 1, the DEGs of each MS stage were identified. The Venn plot of the DEGs of the three MS stages is presented in Fig. 1. Specific information on the upregulation and downregulation of these DEGs can be found in Additional file 1: Table S1, Sheet 1–6. Based on the analysis of differential gene expression in distinct MS stages, 349 DEGs overlapped, among which 196 were upregulated and 149 were downregulated (Additional file 1: Table S1, Sheet 4). Four genes A2M-AS1, LOC102724356, AF520793 and DSERG1 had different dysregulated directions in different disease stages, as shown in Fig. S1. Compared to their expression in the healthy controls, they were all downregulated in the RR-MS stage and upregulated in the PP-MS and SP-MS stages, although their expression levels in the distinct stages were different.

We also tried to obtain DEGs between every two MS stages with the threshold of adjusted FDR adjusted *p* < 0.01 and |log_2_FC| > 1. The specific results can be found in Additional file 1: Table S1, Sheet 7-8. Among these DEGs, the overlap between “SP VS RR” and the 345 common DEGs of the three MS stages were the genes MAP3K8/TPL2, NR4A2 and NAMPT; the overlap between “PP VS RR” and the common DEGs were MAP3K8 and NAMPT. As shown in Fig. S2, MAP3K8 and NAMPT had lower expression levels in MS patients, and their expression levels in the RR-MS group were also significantly lower compared with those in other two MS stages. This suggests that MAP3K8 and NAMPT may be useful markers for differentiating the MS stages.

### GO and KEGG pathway enrichment analysis of DEGs

**Figure 2:**
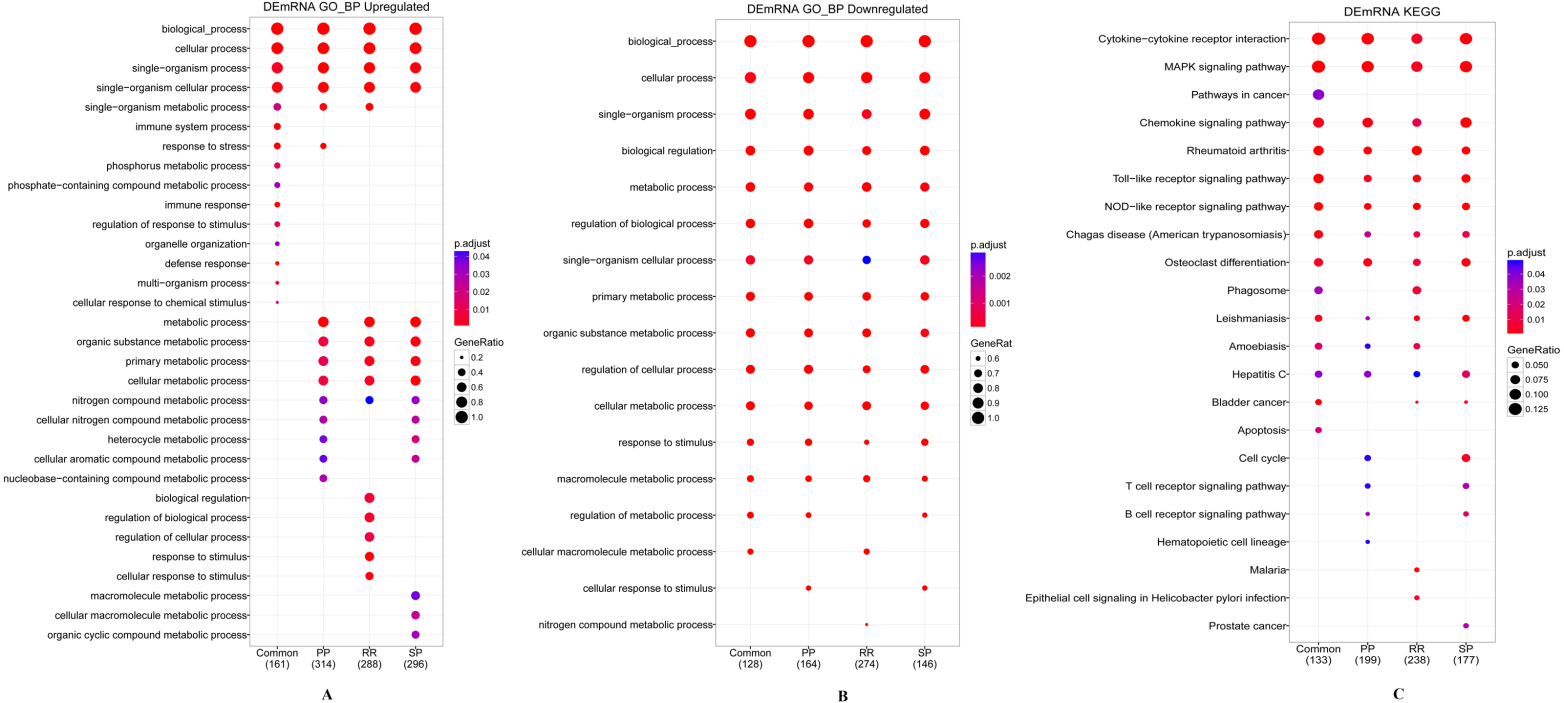
Enrichment analysis results of GO and KEGG. (A) Biological processes analysis of up-regulated DEGs of three MS stages and their common DEGs. (B) Biological processes analysis of down-regulated DEGs of three MS stages and their common DEGs. (C) KEGG pathway analysis of DEGs. The number of genes has the annotation for each set presented in brackets.

The candidate DEGs of each MS stage and their common DEGs were analyzed by their enriched functions and pathways using the R package “clusterProfiler” with a threshold of FDR adjusted *p* < 0.05. The result is shown in Fig. 2, and the specific enrichment results can be found in Additional file 2: Table S2.

For GO, by definition, the subcategories are redundant for their parent categories. As the result list of each group is extensive, redundancy poses great obstacles to the interpretation of the enrichment results and limits the comprehensive comparative analysis of the results of the multigroup enrichment analysis. The R package “clusterProfiler” can aggregate redundant GO categories to produce more concise enrichment results. As shown in Figs. 2A and 2B, the significant GO categories annotated by the downregulated genes of different DEG sets were basically the same, while for upregulated genes, there were some differences. The upregulated genes of the common DEGs were mainly enriched in immune system processes and immune responses alone (Additional file 2: Table S2, Sheet 1). The downregulated genes of the common DEGs were enriched in leukocyte-related processes and inflammatory and immune response regulation processes (Additional file 2: Table S2, Sheet 5). The results of GO enrichment analysis also showed that in the BPs of purine-related metabolic processes and ethanol-related metabolic processes, the upregulated and downregulated genes of the DEG sets of different MS stages were all significantly enriched (Additional file 2: Table S2, Sheet 9). The total specific enrichment results of GO can be found in Additional file 2: Table S2, Sheet 1–9.

The most significantly enriched KEGG pathways of the DEGs are presented in Fig. 2C. The genes of the four DEGs were all enriched in some immune diseases and infectious disease pathways, such as rheumatoid arthritis, Alzheimer’s disease, Parkinson’s disease, hepatitis C, and leishmaniasis. Furthermore, these DEGs were also enriched in signaling pathways associated with the immune system, including the toll-like receptor signaling pathway, MAPK signaling pathway, NOD-like receptor signaling pathway, chemokine signaling pathway, cytokine-cytokine receptor interaction, T cell receptor signaling pathway and B cell receptor signaling pathway (see Additional file 2: Table S2, Sheet 10–13 for the specific enrichment results of KEGG analysis).

### Module analysis and hub gene selection of the PPI network

**Figure 3:**
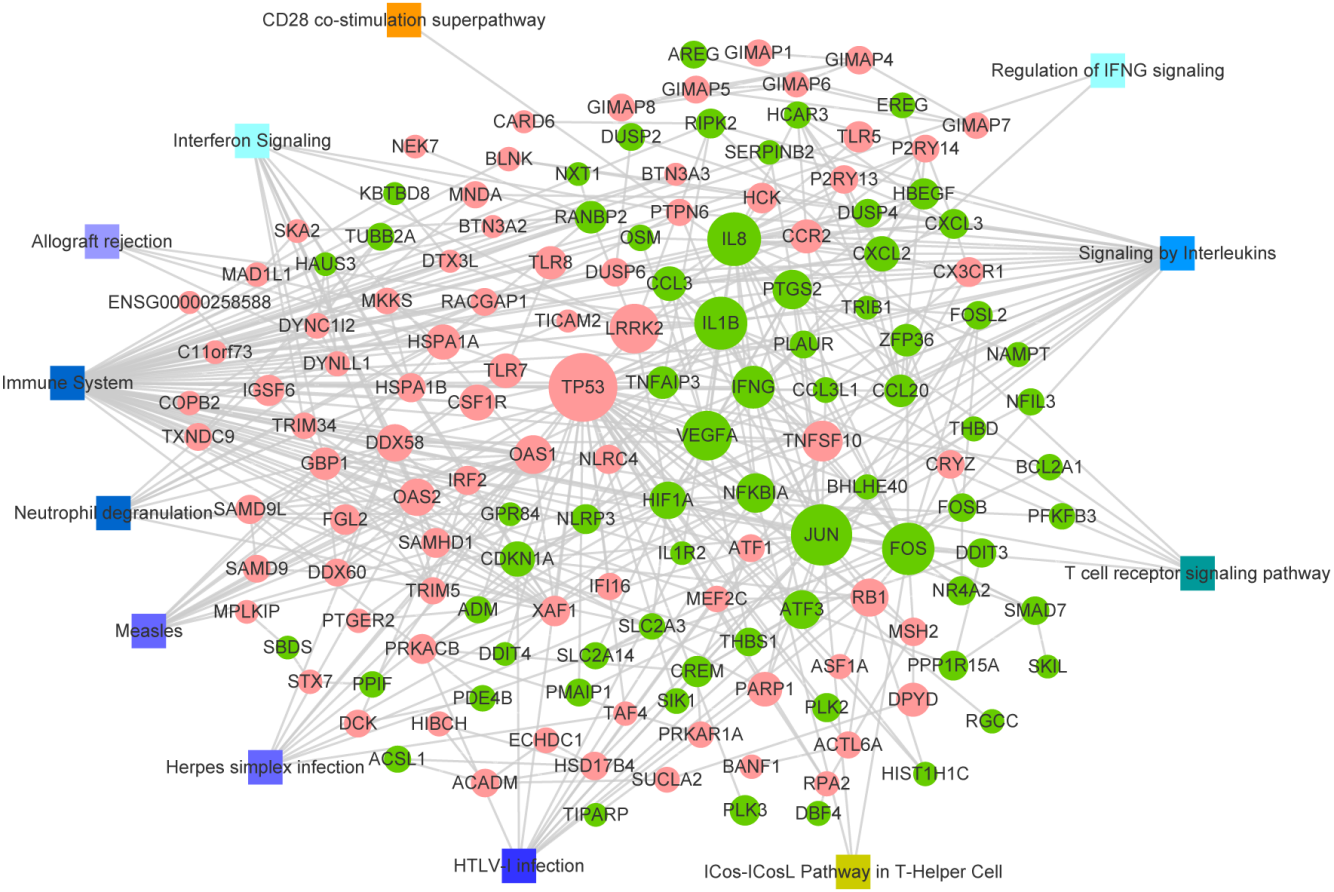
PPI network of common DEGs. Node size indicates the connectivity degree; larger circles indicate a higher degree. Circles indicate genes. Edges indicate the interaction between genes. Red circles, up regulated DEGs; green circles, down regulated DEGs; squares, pathways associated with multiple sclerosis.

Based on the interaction information in the STRING database, we mapped the common DEGs to 908 PPIs with a combined score>0.4. Among them, there were 64 interactions matching the condition of experimentally validation with a score>0.4 (Additional file 3: Table S3, Sheet 1). The PPI network with the interaction of nodes meets the combined score>0.7 is shown in Fig. 3. In addition, the MS related pathways and the nodes of the PPI network involved in them are also displayed in Fig. 3. We have obtained these MS related pathways information through retrieval the database MalaCards: The human disease database (https://www.malacards.org/) which is an integrated database of human maladies and their annotations. As shown in Fig. 3, there are many nodes that involved in the MS related pathway such as Immune System, Signaling by Interleukins, and Interferon Signaling pathway.

In order to identify the significant nodes and hub genes of the PPI networks, CytoHubba a plug-in of Cytoscape was used. The network features scores of each note in the PPI network were calculated by CytoHubba, the results were presented in Additional file 3: Table S3, Sheet 2. As shown in Fig. 3 a larger node indicates a higher Degree. Among the network features of nodes, what we concerned about were Degree and Betweenness. The Degree represents the number of connections or edges of a particular node (Barabasi & Albert, 1999), whereas Betweenness quantifies the flow of information through a node in the network, it specifically shows that a node influences the communication among other nodes (Barabasi & Oltvai, 2004). Here the nodes with higher Degree were considered as significant nodes. We ranked the notes according to their Degree and selected the top seventeen notes with Degree>20 as high connectivity hub genes. We found that the Betweenness of these hub genes almost the highest. Detailed information on these hub genes is shown in Table 1. The heat map of these hub genes based on their expression data is shown in Fig. S3. Obviously, these genes were significantly different between the cases and controls, but the differences were not significant among the distinct stages of MS. Fig. S3 also demonstrated that the genes with similar expression patterns were clustered together.

**Table 1:** Top 17 DEGs of PPI net sorted by Degree.

**Ranks sorted by degree**	**Node_name**	**Degree**	**Betweenness**	**Differentially expressed**
1	TP53	68	12120.51	Up
2	JUN	57	5877.92	Down
3	IL8/ CXCL8	46	3259.12	Down
4	IL1B	45	3568.04	Down
5	FOS	44	2713.07	Down
6	LRRK2	40	7844.68	Up
6	VEGFA	40	1881.45	Down
7	IFNG	30	3053.71	Down
8	TNFSF10	27	1348.96	Up
9	NFKBIA	25	1296.69	Down
9	PTGS2	25	1213.89	Down
10	OAS1	24	1777.25	Up
10	ATF3	24	461.30	Down
11	RB1	23	1328.92	Up
12	OAS2	22	1501.62	Up
12	HIF1A	22	1102.15	Down
12	DDX58	22	966.09	Up

**Figure 4:**
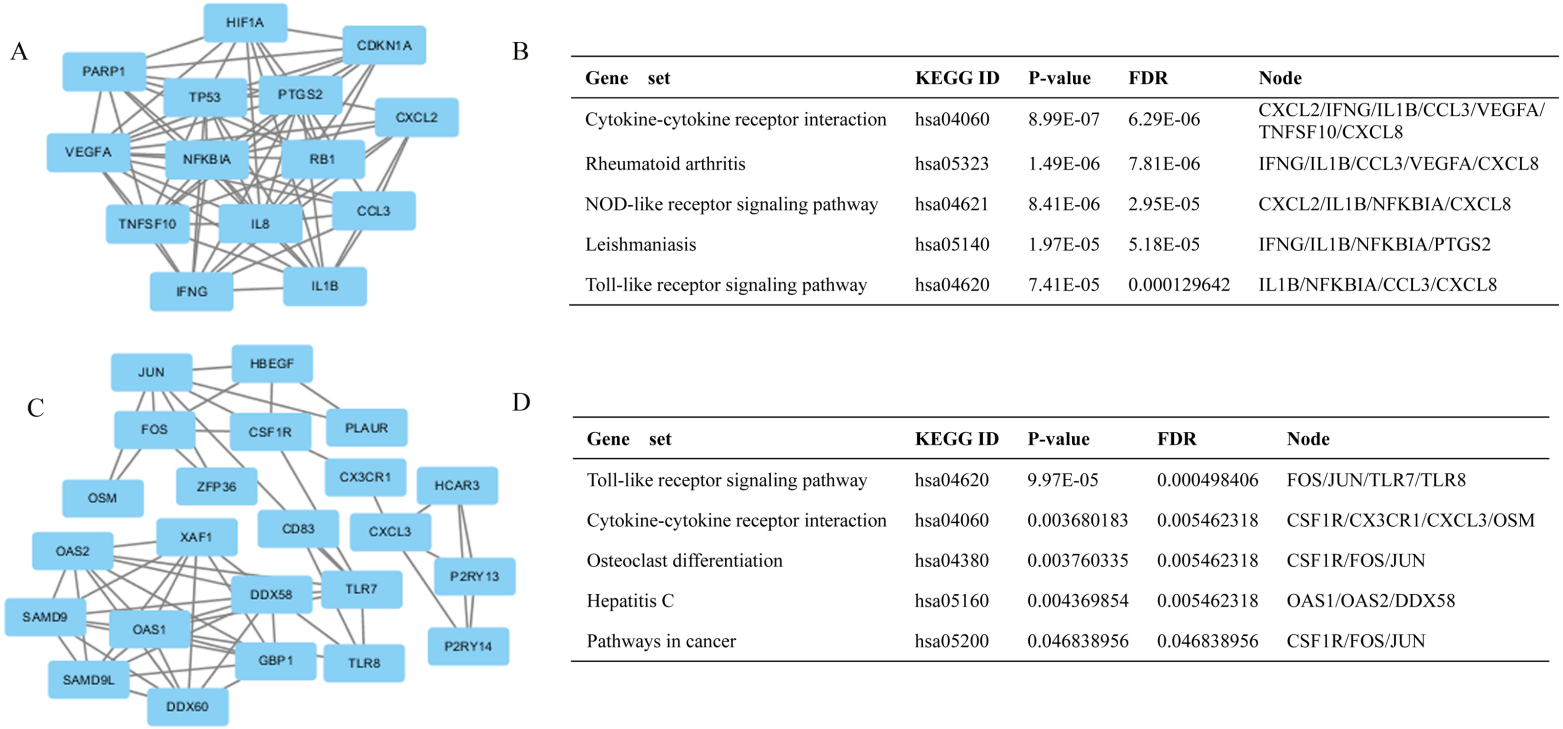
Top two modules from the PPI network and the enriched cellular pathways of module genes. (A) Module 1 and (B) respective enriched pathway. (C) Module 2 and (D) respective enriched pathway.

Additionally, the modules of the PPI network were obtained by using MCODE that a plug-in of Cytoscape. The genes in the network modules are closely related, and they may cooperate to complete the corresponding biological function. Here, we selected the top 2 significant modules and analyzed the KEGG pathways of the genes involved in the modules. The top two models are shown in Figs. 4A and 4C. In Figs. 4B and 4D the enrichment analysis results of genes belong to each model are exhibited. As shown the genes in both modules are significantly enriched in cytokine-cytokine receptor interaction and toll like receptor signaling pathway (FDR adjusted *p* < 0.05). In addition, the genes in module 1 also enriched in Rheumatoid arthritis and NOD-like receptor signaling pathway. In module 2 there are some genes involved in the pathway of cancer. The model genes belonging to each significant pathway are also given in Figs. 4B and 4D. Obviously in both modules there are some genes involved in signaling pathways associated with the immune system. We speculate that the disorder of the corresponding genes in these pathways may lead to the dysfunction of the pathway and lead to the occurrence and development of multiple sclerosis. Moreover, we retrieved information from the ChIPBase and starBase databases to establish the regulatory networks of the hub genes and MS-related miRNAs. The statistics of the related regulatory relationships obtained from these databases are displayed in Additional file 4: Table S4, Sheet 1, and Fig. S4 shows the regulatory networks. In the regulatory network, there are three types of nodes: transcription factor (TF), gene and miRNA. The relationship between nodes represented by edges in the network is activation and inhibition respectively. Here among the 15 hub genes in this regulatory network JUN, TP53, FOS and ATF3 are TFs. These genes play an important role in either the PPI network or the regulatory networks; particularly, JUN, TP53 and FOS have a higher Degree in the regulatory networks (see Table S4, Sheet 2). In addition, the hub genes VEGFA, HIF1A, RB1, PTGS2, CXCL8, OAS2, NFKBIA and OAS1 also have high centrality in the regulatory networks, as shown in Table 2.

**Table 2:** Centrality of hub genes in regulatory networks.

Genes	Degree	Closeness	Betweenness
FOS	50	74.83333	3080.334
TP53	45	73.5	2940.666
VEGFA	40	66	2763.408
JUN	33	68.5	1962.074
HIF1A	32	67.83333	2416.872
RB1	30	63.16667	1282.759
PTGS2	21	57	714.0516
IL8/CXCL8	18	57.16667	536.7006
OAS2	15	48.66667	439.0914
NFKBIA	11	54.25	296.5654
OAS1	10	50.58333	301.027
TNFSF10	7	42.2	30.24196
ATF3	5	47.83333	92.58305
IL1B	1	43.75	0
DDX58	1	43.75	0

### SNP analysis of the common DEGs

By querying the online database SCAN, we retrieved a list of SNPs that were significantly associated with the expression of the 345 common DEGs of the MS stages. Then, we searched these SNPs on the MirSNP database and obtained 6857 correlation pairs of these SNPs, miRNAs and genes (Additional file 5: Table S5, Sheet 1). There were a total of 1697 miRNAs in these correlation pairs. Then, we contrasted these miRNAs with 149 MS-related miRNAs extracted from the following databases: HMDD v2.0, miR2Disease and MNDR v2.0 (Additional file 5: Table S5, Sheet 2). Of these 1697 miRNAs, 115 miRNAs were found to be MS-related, and they were mapped to 467 correlation pairs that were composed of 156 DEGs and 321 DEGs’ eSNP (Additional file 5: Table S5, Sheet 3). Moreover, of these 156 genes, four genes were related to MS by GWAS (GWASdb: http://jjwanglab.org/gwasdb). Fifteen SNPs were identified to be associated with these 4 genes, which in turn were controlled by MS-specific miRNAs. This strengthens the association of these 15 SNPs with MS (Table 3).

**Table 3:** Specific SNPs with their associated MS related miRNAs and genes.

**microRNAs**	**SNPs**	**Chromosome**	**Genes**
hsa-miR-142-3p	rs34396430	6:31830173	HSPA1B
hsa-miR-548am-3p	rs9267570	6:31830142	HSPA1B
hsa-miR-505-5p	rs1049479	1:192579386	RGS1
hsa-miR-22-5p	rs2816308	1:192579429	RGS1
hsa-miR-31-5p	rs3829148	10:6233821	PFKFB3
hsa-miR-31-5p	rs36102318	10:6233809	PFKFB3
hsa-miR-31-5p	rs55643411	10:6233813	PFKFB3
hsa-miR-376a-3p	rs35781052	10:6234072	PFKFB3
hsa-miR-335-5p	rs1064893	10:6234731	PFKFB3
hsa-miR-449b-5p	rs1064898	10:6234972	PFKFB3
hsa-miR-34a-5p	rs1064898	10:6234972	PFKFB3
hsa-miR-922	rs11541589	10:6234547	PFKFB3
hsa-miR-4677-3p	rs3814190	10:6234757	PFKFB3
hsa-miR-204-5p	rs1054519	12:57516605	DDIT3
hsa-miR-376a-3p	rs1054519	12:57516605	DDIT3

Through querying the GWASdb, we obtained 1010 MS-related SNPs (Table S5, Sheet 4). Combined with the MS-related miRNAs obtained above, we queried the MirSNP database and found that there were 3 MS-specific correlation pairs, as shown in Table 4. Though neither of the two genes is differentially expressed in our data set, the SNP and miRNA associated with the two genes are all MS-related.

**Table 4:** MS related SNPs and miRNAs along with their associated genes.

**microRNAs**	**SNPs**	**Chromosome**	**Genes**
hsa-miR-181b-5p	rs4819388	21:44227538	Inducible T cell costimulator ligand (ICOSLG)
hsa-miR-24-3p	rs4819388	21:44227538	Inducible T cell costimulator ligand (ICOSLG)
hsa-miR-21-3p	rs3177928	6:32444658	Major histocompatibility complex, class II, DR alpha(HLA-DRA)

## Discussion

At present, the cause of MS has not been fully elucidated, but it is believed to involve bacterial induction and/or environmental and genetic factors, of which genetic factors are essential to determining the process of MS occurrence and development. When triggered by environmental factors or bacterial induction, multiple genes may influence a person’s chance of developing MS (Hemmer, Kerschensteiner & Korn, 2015).

In the present study, we reused the expression profile data of a previous study by Srinivasan et al., their study had provided evidence that GWAS genes (genes located in the vicinity of MS risk variants) display dysregulated expression in peripheral blood of subjects with distinct MS stages compared with healthy individuals, and those genes contribute to pathogenic pathways in MS. The previous study was focused on GWAS genes, the dysregulated condition of these genes at different MS stages and their roles in MS pathogenic pathways were studied. The results of previous study indicated that alteration in the expression of MS GWAS genes is not a stable feature reproduced throughout the distinct phases of disease, but is a selective event for each disease stage. It also demonstrated that only the GWAS gene CD86 was consistently overexpressed in PBMC at all the stages of MS compared with health controls in both discovery set and validation set. In our study, we reused the dataset E-MTAB-5151 to identify the potential key genes of MS. Firstly we identify the genes with significant and consistent dysregulation in each MS stage through differential expression analysis. A total of 345 common DEGs (193 upregulated and 117 down-regulated genes) were obtained through differential expression analysis. There were no differentially expressed genes that met our thresholds of FDR adjusted *p* < 0.01 and |log_2_FC| > 1 between patients in the PP-MS and SP-MS stages. This result suggests that despite the clear differences in the clinical symptoms of MS patients in the two stages, most of the genes have relatively similar expression levels. We also found that the expression levels of MAP3K8 and NAMPT were significantly different between the RR-MS stage and the other two MS stages. MAP3K8 is a mitogen-activated protein kinase (MAP3K) that is activated downstream of TNF *α*R, IL1R, TLR, CD40, IL17R, and some GPCRs. It regulates the MEK1/2 and ERK1/2 pathways to regulate a cascade of inflammatory responses. MAP3K8 activates p38 *α* and p38 *δ* to drive the production of various inflammatory mediators in neutrophils (Xu et al., 2018). The protein encoded by NAMPT is thought to be involved in many important biological processes, including metabolism, stress response and aging. It can participate in the development of inflammatory diseases, metabolic diseases and chronic kidney disease. We speculate that the differential expression of these two genes may lead people to become susceptible to MS, and their expressed level may be associated with the stage of MS susceptibility.

Through the GO enrichment analysis of DEGs, we found that the common DEGs were significantly enriched in some immunological process, and the DEGs of different MS stages were all significantly enriched in BPs of the purine-related metabolic process and signaling, adenylate cyclase-activating G-protein coupled receptor signaling pathway, regulation of adenylate cyclase activity, ethanol oxidation and ethanol metabolic process, etc. Accumulating evidence has indicated that alterations in purinergic system signaling are involved in immunity and inflammation. Extracellular adenosine directly affects various physiological and pathological processes of MS by stimulating the G protein-coupled adenosine receptors A1, A2A, A2B, and A3 on the surface of immune cells (Safarzadeh et al., 2016; Mayne et al., 1999; Sperlagh & Illes, 2007). Additionally, some studies have shown that alcohol has both immunomodulatory and neuroprotective properties and may exert an effect on the disease course of multiple sclerosis (MS) and other autoimmune diseases such as systemic lupus erythematosus and rheumatoid arthritis (Diaz-Cruz et al., 2017; Noble & Weimer, 2014; Nova et al., 2012). In addition, the DEGs were mainly enriched in pathways associated with inflammation and the susceptibility to immune diseases. These findings were in accordance with the well-established conclusion that MS is a kind of autoimmune diseases it is characterized by autoimmune response and inflammation.

To understand the related interactions among the identified common DEGs of the MS stages, we established a PPI network. Seventeen genes with Degree greater than 20 were identified as hub genes, including TP53, JUN, IL8, FOS, VEGFA, NFKBIA, PTGS2, OAS1, RB1, OAS2, HIF1A, IL1B, LRRK2, IFNG, TNFSF10, ATF3 and DDX58. Among these genes, some were upregulated and others were downregulated in MS patients. We did cluster analysis with the expression level of these seventeen hub genes as characteristics on the 76 samples (49 MS patients and 27 healthy controls) in our study, and the result is shown in Fig. S5A. Obviously the MS patients and Healthy Controls (HC) were classified successfully. In addition, for verification we used another PBMCs expression profile data that also downloaded from EBI Arrayexpress database (Accession number E-GEOD-21942). This dataset was obtained from 10 MS patients and 15 healthy unrelated controls. The same hierarchical cluster method was used with the expression level of the seventeen hub genes as characteristics. The Cluster dendrogram is shown in Fig. S5B, the MS patients and HCs were well separated. These results demonstrate that these seventeen hub genes we identified can be used as potential biomarkers for establishing MS diagnostic model. Then, we constructed a regulatory network with these hub genes and MS-related miRNAs to further screen the key candidate genes and explore the potential gene expression regulatory mechanisms associated with MS. Finally, we found that FOS, TP53, JUN, VEGFA, HIF1A, RB1, PTGS2, IL8, OAS2, NFKBIA, and OAS1 are not only the ‘network-hub’ genes that interact with many of the DEGs of MS patients but also the central genes involved in the regulatory network with MS-related miRNAs. Among these genes, TP53, FOS, JUN and ATF3 are transcription factors. TP53 is a well-known immunosuppressor gene that is upregulated by proinflammatory cytokines and is implicated in MS severity (Meek, 2015; Giacalone et al., 2015). The aberrant activation of JUN may result in the inhibition of T cell activation (TCA), and since MS is a chronic debilitating disease of the central nervous system, it is primarily mediated by T lymphocytes with specificity to neuronal antigens in genetically susceptible individuals (Baer, Colon-Moran & Bhattarai, 2018). FOS is a neuronal activity-associated transcription factor. The FOS gene family encodes leucine zipper proteins that can dimerize with proteins of the JUN family, thereby forming the transcription factor complex JUN.

For the other key candidate genes, we found that their function had some association with immunity, inflammation, neural activity, cell proliferation, and cell cycle regulation. For example, the protein encoded by the IL8 gene is a member of the CXC chemokine family, is a major mediator of the inflammatory response and is also a potent angiogenic factor. It was shown that at diagnosis, increased levels of IL8 are detected in the cerebrospinal fluid (CSF) of MS patients with higher levels of gray matter damage (Carvalheiro et al., 2018; Magliozzi et al., 2018). Vascular endothelial growth factor A (VEGFA) is a member of the PDGF/VEGF growth factor family. It stimulates angiogenesis, is also proinflammatory and plays an important role in the development of neurological disease. Studies have shown that there is an association between vascular endothelial growth factor-related factors and the severity of multiple sclerosis  (Kouchaki et al., 2016; Chapouly et al., 2015). NFKBIA-encoded proteins interact with REL dimers to inhibit NF-kappa-B/REL complexes, which are involved in inflammatory responses and control a variety of immune-related genes (Zang et al., 2002). The proteins encoded by OAS2 are essential proteins involved in the innate immune response to viral infection; OAS2 is an interferon-regulated gene and its proteins are recurrently proposed in the literature as predictive biomarkers of interferon-beta treatment response (Martire et al., 2016). Additionally, there are some studies on the susceptibility variant in OAS1 for MS  (Cagliani et al., 2012; O’Brien et al., 2010; Fedetz et al., 2006). MS is a chronic inflammatory demyelinating disease of the CNS with evidence of immune dysfunction, and based on the above analysis of the key candidate gene functions, we believe that some of these key candidate genes may be useful biomarkers for diagnosing MS and predicting relapses in MS patients.

In our study, based on the common DEGs of different MS stages, we used an online database to mine potential SNPs associated with MS. The identified candidate SNPs are significantly associated with the expression of DEGs; simultaneously, these SNPs are all located at the binding site of MS-related miRNAs to DEGs. In addition, we found that rs4819388 and rs3177928 are MS-related SNPs in GWASdb. Specifically, rs4819388 is on the binding site of ICOSLG with MS-related miRNA hsa-miR-181b-5p and hsa-miR-24-3p, and rs3177928 is on the binding site of HLA-DRA to hsa-miR-21-3p (Table 4), which is also MS related. Both ICOSLG and HLA-DRA are known to be involved in immunity, and considering that MS is a type of autoimmune disease, perhaps this relationship explains the mechanism of rs4819388 and rs3177928 in MS and indicates that ICOSLG, HLA-DRA may be associated with MS.

It is worth noting that there were 24,442 genes of the HG-U133_Plus_2 Array after removing redundancy, among which there were 345 common DEGs of the three MS stages. We collected the correlation pairs of 149 MS-related miRNAs using the MirSNP database. There were 13,860 genes in these pairs after removing redundancy, among which 12879 genes overlapped with those in the HG-U133_Plus_2 Array, including 245 common DEGs of MS. We performed a hypergeometric test, and the *p* value was 1.138357e−12, which indicates that the genes of the MS-related miRNA correlation pairs from the MirSNP database are enriched in the common DEGs of the three MS stages. Thus, the SNPs and regulatory relationships identified on the basis of the MirSNP database and MS-related miRNA may have biological significance in MS progression, which deserves further study.

In conclusion, we combined gene expression profile data with bioinformatics analysis and identified key genes, SNPs, pathways and regulatory relationships that may be involved in the occurrence and development of MS. The present study may provide a basis for an improved understanding of MS and may be helpful for further MS clinical research. However, our study has some limitations of data sources and statistical methods, and the current findings lack experimental verification *in vivo* and vitro. Therefore, future functional studies should be conducted to confirm the expression and function of the identified genes and validate the regulatory relationship among the specific molecules.

## Supplemental Information

10.7717/peerj.8357/supp-1 Figure S1 Box figure of expression condition of four special genes A2M-AS1, LOC102724356, AF520793 and DSERG1

10.7717/peerj.8357/supp-2 Figure S2 Box figure of expression condition of genes MAP3K8, NAMPT and NR4A2

10.7717/peerj.8357/supp-3 Figure S3 Heat map of the hub genes

10.7717/peerj.8357/supp-4 Figure S4 Regulatory network of the hub genes. Circles indicate genes, triangles transcription factors, and V-shapes indicate miRNA. For the hub genes green means up-regulated and red means down-regulated Circles indicate genes, triangle s transcription factors, and V-shapes indicate miRNA. For the hub genes green means up-regulated and red means down-regulated.

10.7717/peerj.8357/supp-5 Table S1 Results of differential expression analysis

10.7717/peerj.8357/supp-6 Table S2 Results of enrichment analysis

10.7717/peerj.8357/supp-7 Table S3 Attributes of the PPI network

10.7717/peerj.8357/supp-8 Table S4 Node attributes of net of MS related miRNA and hub genes

10.7717/peerj.8357/supp-9 Table S5 Results of SNP analysis of multiple sclerosis

10.7717/peerj.8357/supp-10 Figure S5 Cluster dendrogram (A). The cluster dendrogram of E-MTAB-5151 dataset; (B). The cluster dendrogram of E-GEOD-21942 dataset. The Chebyshev distance was adopted and the method for calculating the distance between classes was the average linkage. HC: Healthy control; MS: Multiple sclerosis.

## Additional Information and Declarations

### Data Availability

The following information was supplied regarding data availability: Data is available at the ArrayExpress database: E-MTAB-5151, E-GEOD-21942.
