# Supplementary figures and images for: Identification of key genes associated with multiple sclerosis based on gene expression data from peripheral blood mononuclear cells
Source: PeerJ. 2020 Feb 3;8:e8357. doi: 10.7717/peerj.8357 (PMC7003695; doi:10.7717/peerj.8357)

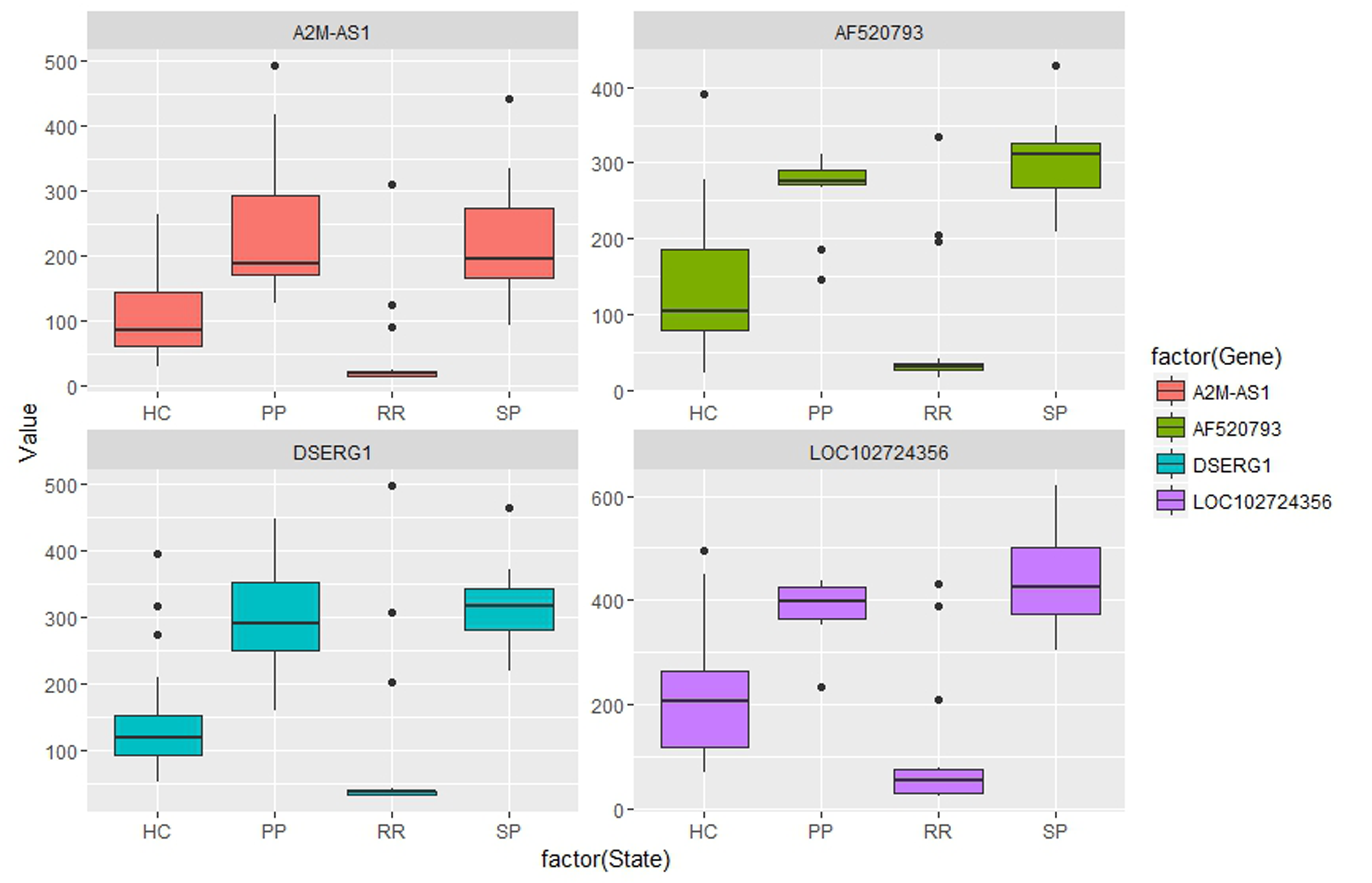

Supplement: Figure S1 [file peerj-08-8357-s001.png]

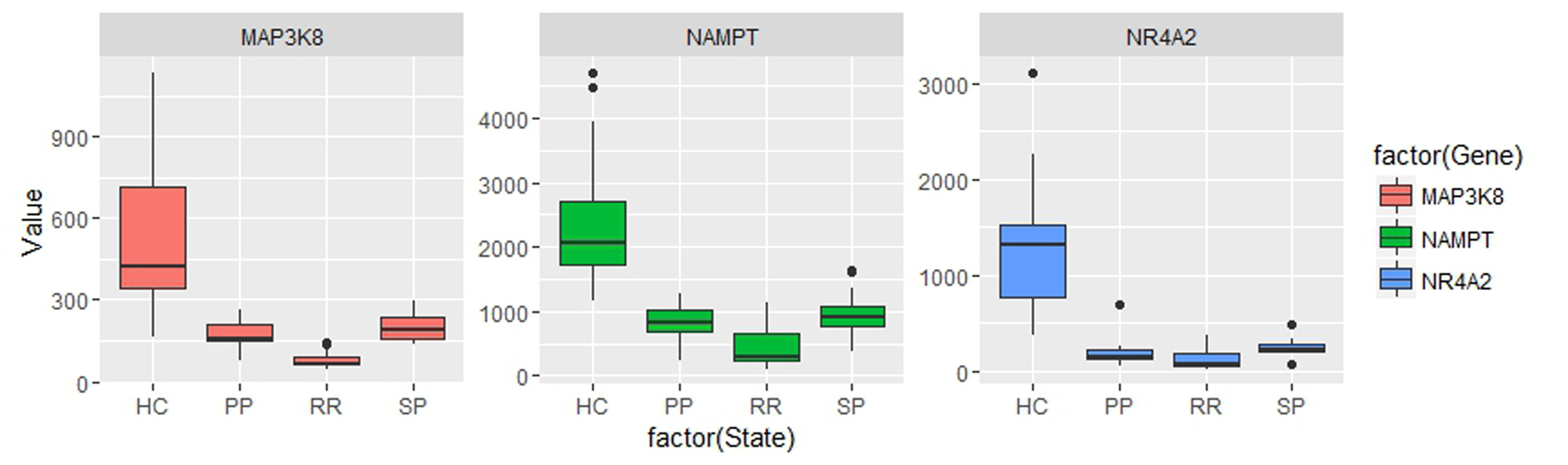

Supplement: Figure S2 [file peerj-08-8357-s002.png]

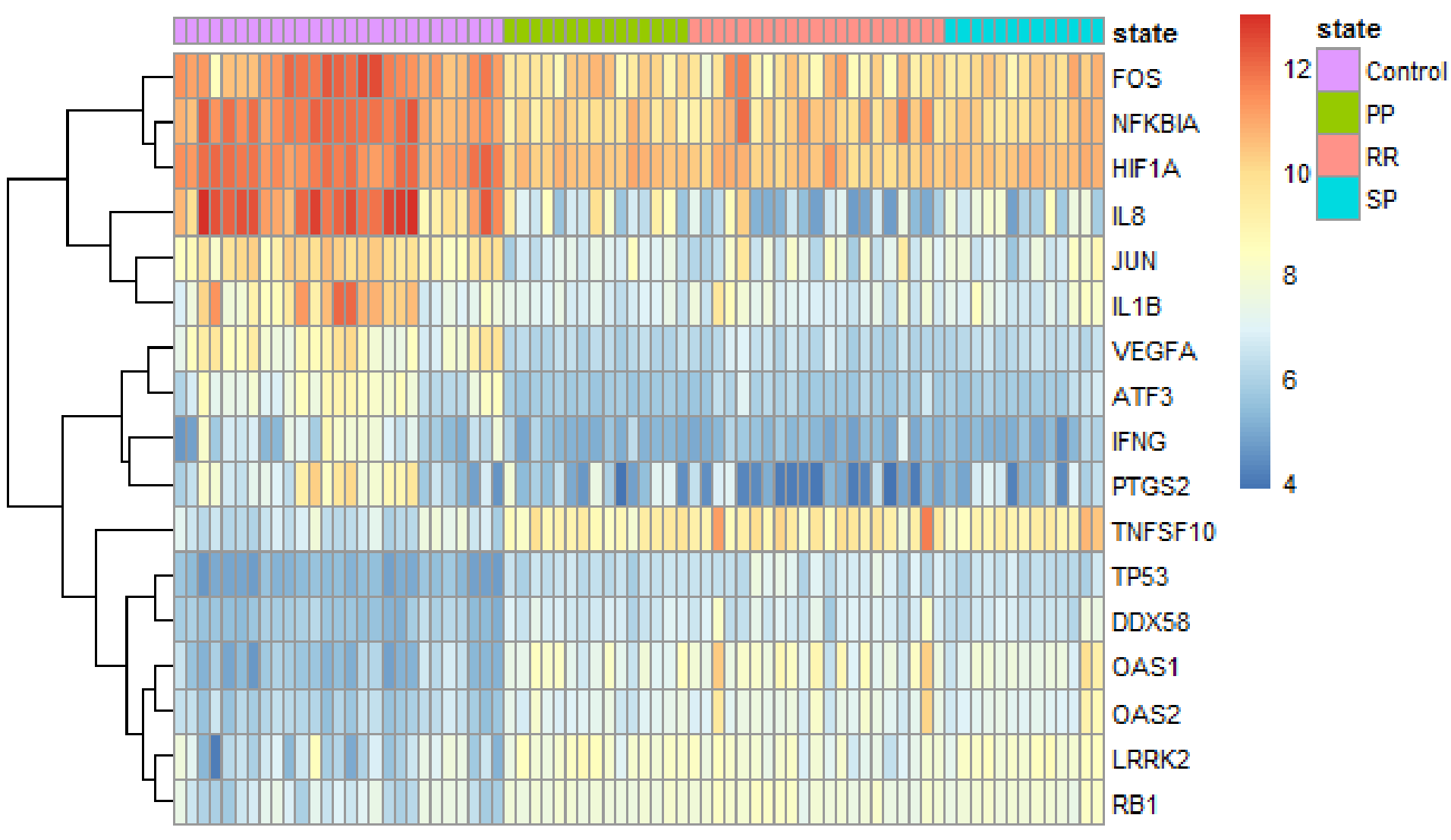

Supplement: Figure S3 [file peerj-08-8357-s003.png]

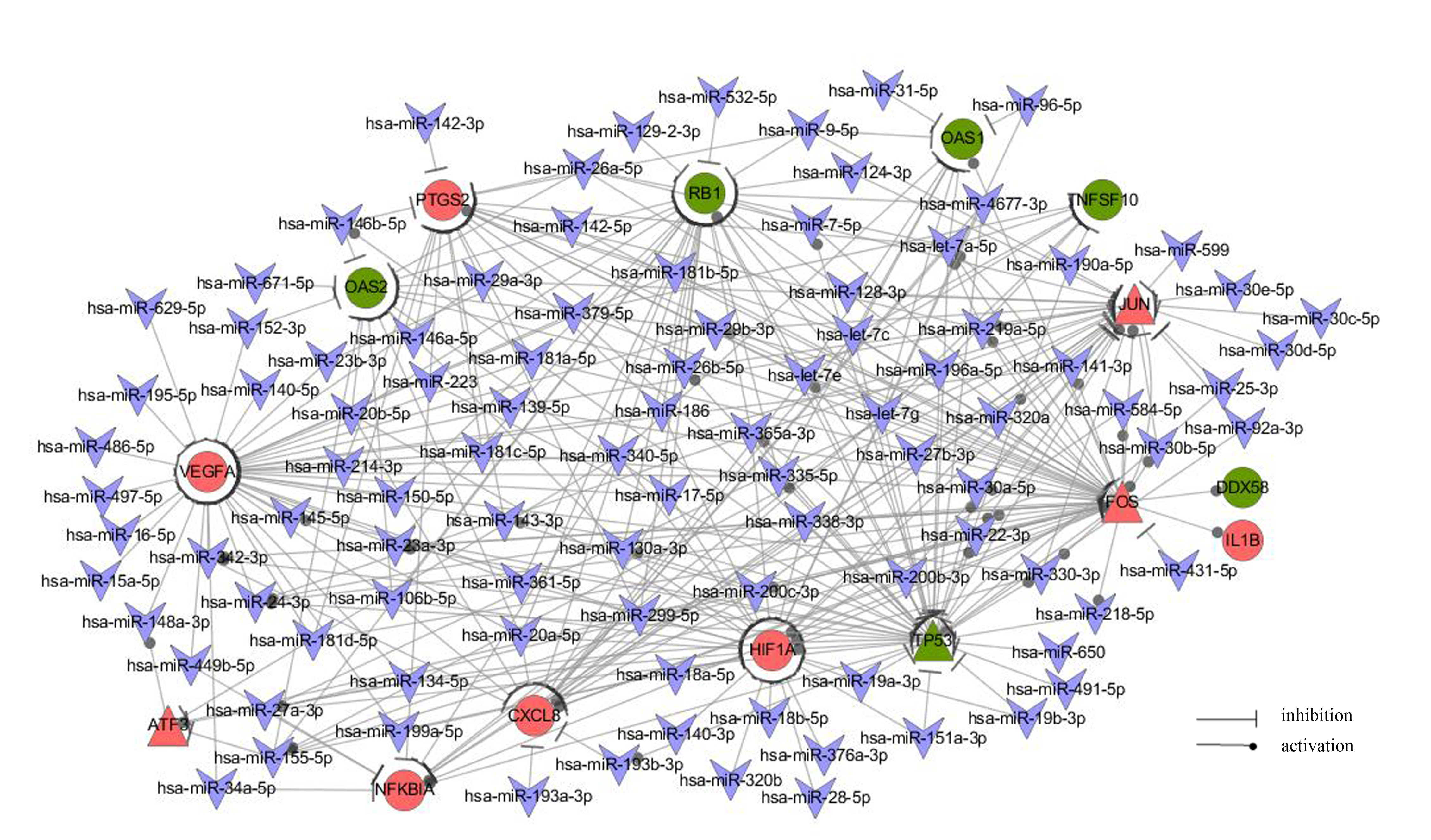

Supplement: Figure S4 — Circles indicate genes, triangle s transcription factors, and V-shapes indicate miRNA. For the hub genes green means up-regulated and red means down-regulated. [file peerj-08-8357-s004.png]

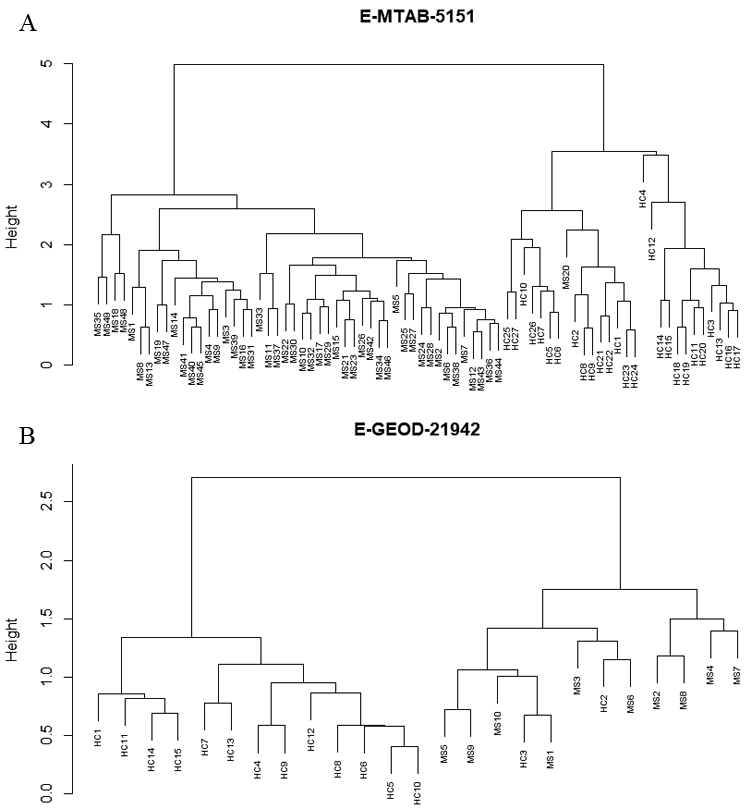

Supplement: Figure S5 — (A). The cluster dendrogram of E-MTAB-5151 dataset; (B). The cluster dendrogram of E-GEOD-21942 dataset. The Chebyshev distance was adopted and the method for calculating the distance between classes was the average linkage. HC: Healthy control; MS: Multiple sclerosis. [file peerj-08-8357-s010.png]
